# The Relationship Between Biomechanical-Anthropometrical Parameters and the Force Exerted on the Head When Heading Free Kicks in Soccer

**DOI:** 10.5812/atr.5307

**Published:** 2012-06-01

**Authors:** Meghdad Teymouri, Heydar Sadeghi, Amir Nabaei, Amir Kasaeian

**Affiliations:** 1 Department of Physical Education, Shahrood Branch, Islamic Azad University, Shahrood, IR Iran; 2 Department of Physical Education and Sport Sciences, Tarbiat Moallem University, Tehran, IR Iran; 3 Department of Electrical Engineering, Shahrood University of Technology, Shahrood, IR Iran; 4 Department of Epidemiology and Biostatistics, School of Public Health, Tehran University of Medical Sciences, Tehran, IR Iran

**Keywords:** Soccer, Head, Force

## Abstract

**Background:**

Soccer is a contact sport in which the players are frequently faced with the risk of injury. It has been shown that the force exerted on the head during heading can be as much as 500–1200 Newton (N).

**Objectives:**

The main objective of this study was to determine whether there was any relationship between the force exerted on the head and several biomechanical and anthropometrical parameters related to heading free kicks.

**Patients and Methods:**

A total of 16 semi-professional soccer players with at least 5 years experience participated in this study. The mean age, height, and weight of the study participants were 21.36 ± 5.67, 178 ± 5.99 cm, and 70.55 ± 8.55 kg, respectively. To measure the force exerted on the heads of the players, a pressure gauge was installed on their foreheads. Each participant was asked to defend the ball using the heading technique three times. A camera with a sampling frequency of 150 frames per second was used to record the moment of impact between the ball and head during each heading event. For each participant and replicate, the ball and head velocity (m/s) as well as the angular body changes (degrees) were calculated using MATLAB and AutoCAD softwares, respectively. Descriptive statistics, including means and standard deviations were used to describe the data. Pearson correlation coefficient (alpha = 0.05) was used to examine potential relationships between the variables of interest.

**Results:**

Significant correlations existed between the force exerted on the head during heading, participant age, body mass, body fat percentage, and head perimeter (*P *< 0.05).

**Conclusions:**

The study revealed the significance of anthropometric variables related to heading, such as age and head perimeter. Therefore, it was concluded that these variables should be considered when teaching and practicing the heading technique with players of different ages and anthropometric sizes.

## 1. Background

Soccer is one of the most popular sports in the world. According to reports from the World Football Federation, 250 million people play soccer regularly and this number continues to increase (1). One of the factors widening the appeal of this sport in the world is the use of different techniques that utilize different parts of the body to carry or shoot the ball during competition. Heading is a versatile technique used by players in both attacking and defensive play. Using this technique requires appropriate practice and coordination of different parts of the body when the ball strikes the forehead. Heading is typically initiated by the extension of the back, in which the upper part of the body moves forward. During this movement, the head, neck, and trunk act in coordination while the chin is in front of the chest. The backward movement of the body gives a player the ability to move forward (to head) at a higher speed. The strength and power behind heading is attributed to the movement of the trunk and the femoral flexors (2), while the hands and legs are generally used to protect the body, depending on the player’s skill level and technique.

Frengueli *et al*. (1991), who studied the injuries of people receiving therapy, found that of the 208 injuries investigated, 22.7% involved the neck and head and 65% had occurred while playing soccer. In another study, Tysvaer (1992) reported that soccer-related injuries to the head and neck ranged from 4% to 22%; however, there is no consensus on the major cause of these injuries. Mehnert *et al.* (2005) studied the technique of heading from different perspectives and noted that if the proportion of the mass of the head to the mass of the ball decreases, the force exerted on the head and the risk of injury to the head will increase. By studying the speed of the head in heading, Tyranni *et al.* (2008) came to the conclusion that among several variables, including the “perimeter of the head,” the “strength of flexor and extensor muscles,” and the “mass and length of the head and the neck,” the mass of the head played the most important role in decreasing the acceleration of the head during heading. In most cases of heading, the ball is traveling at an average speed of 18.05 m/s. The impact of a ball traveling at 10.23 m/s will produce 812–815 N of energy and a velocity of 30–55 g (6). Monto *et al.* (2002) reported this level of force to be 500–1200 N (8). Ziejewski (2000), using a computer-simulated model, concluded that the impact of the ball on the head produces a force of 150–200 pounds (666–888 N); however, this is less than estimates calculated from real data. Importantly, a brain injury resulting from ball-to-head contact can occur at a force of 400–1000 N (7,9).

## 2. Objectives

Given that heading is one of the primary techniques used in soccer during which the player undergoes impacts to the head, in the present study we investigated potential relationships between the force exerted on the head during heading and heading-related biomechanical and anthropometric parameters.

## 3. Patients and Methods

Sixteen soccer players, with at least 5 years experience as semi-professional players, participated in this study as subjects. At the time of study, all of the players were members of semi-professional league teams and participated in three to five sessions of specialized practice every week. The mean age of the players was 17.5 ± 1.93 years, and their average height and body mass were 177.08 ± 6.54 cm and 63.88 ± 4.95 kg, respectively. This study was approved by departmental and university regulatory committees. After making the appropriate arrangements with the participating teams, the goals and purpose of the study were explained to the players, and participants were required to complete and signing formed consent documents. Anthropometric measurements were carried out using the appropriate equipment. Briefly, we used a meter tape for measuring head height and perimeter, and a mechanical scale with the accuracy of 100 g was used for measuring body mass and strength of the neck muscles. The mechanical scale was installed and positioned on the wall based on the height of the study participants. We used a skinfold caliper with an accuracy of 1 mm for measuring subcutaneous fat. All biomechanical data were collected in the penalty area of a soccer pitch. Players were asked to stand in a 6 × 6 m^2^ area and defend three free kicks only using their heads. To measure the force exerted on the head during heading trials, a pressure sensor, which could measure the gas pressure of one atmosphere, was attached to a pressure gauge apparatus on the foreheads of the participants. Pressure gauges were set by placing the sensor in an air bag and adjusting the pressure inside the bag using a small pump. The air pressure in the pressure gauge was set in a way that only the pressure exerted on the gauge could be measured and the direct contact of the ball with the forehead would be prevented. Data from the pressure gauge was transmitted to a computer via a wireless system at a rate of approximately 300 samples per second, and data was saved only for trials in which pressure readings had been registered in the computer and the related scene had been filmed. In order to collect motion data of the ball and header for each trial, we used a Kodak camcorder with a recording rate of 150 frames per second. The camcorder was installed 10 m from the participants at a height of 2.5 m. The angle of the camcorder was set to 90º such that we could record the movement of the soccer ball.

For measuring the percentage of body subcutaneous fat we used the four-site system (biceps, triceps, subscapular, suprailiac) and the formulas incorporated in the booklet for the caliper. The mass of participants’ head was calculated using the following formula (9):

Mass of the head = 0.023× (mass of the body in Newtons) + 18.70

In order to confirm the validity of readings registered by the computer and the pressure inside the airbag, acquired pressure readings were calibrated using readings collected using a standardized pressure gauge. Using the Pearson product-moment correlation coefficient, the collected data were found to be highly correlated to the readings generated by the standardized pressure gauge (r = 0.9), indicating the validity of the pressure gauge. The reliability of the pressure gauge was tested further using the test-retest method with three replicates. The mean of the obtained correlation coefficients for relationships between the first, second, and third replicates, was r = 0.6.

To convert pressure data to N, the gauge was set to the inactive mode. We then exerted pressure on the gauge using a soccer ball, and the kg values were recorded. This procedure was repeated, and the readings obtained from the pressure gauge (x) were converted to N using the following formula:

F (Newton) = (-62 + [(0.1 × x) ×8]) × 10

 The information collected with the camcorders was processed using Matlab R2008 (Version 7.6.0) using the extension “mov.” Pixels (measuring unit of the software) were converted to m using the estimated size of the ball in each image (based on the number of pixels filling the image of the ball) and the actual size of the ball in m. Two points were fixed near the point of impact between the ball and forehead. From the coordinates of the two fixed points, the movement of the ball was calculated. Having obtained the time the ball took to move a given distance, the velocity was then calculated using the following formula:

v = x / t

The momentum of the ball and head was measured by multiplying their mass by the velocity. It should be noted that the approximate mass of the ball was 0.3 kg. Images capturing the impact of the ball and the head were loaded into AutoCAD 2007. Anatomic parts of the body, including the ear auricle, acromion process, and the iliac crest were determined and linked together. The angle of the head (the angle between the line created by the auricle, the acromion process, and the line perpendicular to it) and trunk (the angle between the line created by the acromion process, iliac crest, and the line perpendicular to it) were then measured. Data analyses were conducted using SPSS 16. We used descriptive statistics to classify and categorize the data and to determine the arithmetic means and standard deviations. We also used inferential statistics (the Pearson Product Moment Correlation) to determine the correlation between the variables at a 95% level of significance.

## 4. Results

Our results show that 17.9% of the participants headed free kicks without jumping, 43.6% of them headed by jumping in place, and 38.5% of them headed the ball by jumping forward. The means and standard deviations for the variables related to the speed of the ball and the head both before and after heading are shown in Table 1. Our results showed that the speed of the head and the speed of the ball decreased 38.85% and 6.07% proportionally. The mean, standard deviations, and correlation coefficients for relationships between biomechanical-anthropometric parameters and the force exerted on the participants’ heads when heading free kicks are presented in Table 2.

**Table 1.** The Means and Standard Deviations for Variables Related to Speed

	The Ball Speed, Mean ± SD	The Ball Momentum, Mean ± SD	The Head Speed, Mean ± SD	The Head Momentum, Mean ± SD
Before heading	17.99 ± 2.36	5.40 ± 0.71	3.61 ± 1.51	7.4 ± 4.97
After heading	15.93 ± 3.14	4.59 ± 1.35	1.59 ± 0.82	5.95 ± 3.03

**Table 2.** Correlation Coefficients for Variables

	The Free-Kicks		
	Mean ± SD	Correlation Coefficient	Significance
Ball speed, m/s	17.99 ± 2.36	0.18	0.29
Head speed, m/s	3.61 ± 1.51	0.08	0.65
Angle of the head	59.94 ± 20.17	-0.29	0.11
Angle of the trunk			
Flexion	14.32 ± 12.01	-0.17	0.43
Extension	22.67 ± 9.41	-0.19	0.62
Strength of neck flexor muscles, kg	12.41 ± 2.95	-0.11	0.51
Head perimeter	56.37 ± 1.87	-0.50^b^	0.00
Percentage of subcutaneous fat	12.29 ± 3.34	-0.39^a^	0.02
Body mass, kg	67.66 ± 8.49	-0.38^a^	0.02
Age, y	20 ± 5.17	-0.43 ^b^	0.01

^a^Significant Correlation (P ≤ 0.05)

^b^Significant Correlation (P ≤ 0.01)

## 5. Discussion

The purpose of this study was to investigate potential correlations between the force exerted on the head during free kicks and a number of biomechanical/anthropometric variables: age, joint angles and the strength of extensor neck muscles, ball speed, head speed, head perimeter, percentage of subcutaneous fat, and body mass. Our results showed that the force exerted on the head of the soccer players during heading was 685–1100 N. These findings were consistent with previous studies (7, 11, 12), and because our data were collected close to the start of competition, it is likely that our results closely represent reality. We found no evidence for a significant correlation between the speed of the ball and the force exerted on the head when heading (*P* = 0.29). In this respect, our results were not consistent with the results of Naunheim *et al.* (2003) who reported that higher ball speeds were associated with greater momentum and force exerted on the head when heading. Future studies are therefore needed to further evaluate the relationship between the velocity of the ball and the force exerted on the head of player. Our results also showed that there was not a significant correlation between the speed of the head and the force exerted on the head during heading (*P* = 0.65). No previous investigations, to our knowledge, have considered these factors; therefore, we could not compare our results with those of other studies. The findings related to the angles of the joints in the upper limb were not consistent with the results of Sunami *et al.* (2008). This inconsistency is probably the result of the use of different methods to measure and determine the angle of the joints. In the present study, although the points used to characterize the angles of the joints were similar to those in Sunami *et al.* (2008), we considered only the joints on one side of the body, whereas Sunami *et al.* (2008) used measurements from joints on both sides of the body, and linked them together. In addition, statistical analyses showed that there was no significant correlation between the angles of the head and flexion of the trunk, and the force exerted on the head when heading free kicks (*P *> 0.05). On the basis of these results, it seems that heading technique does not play a significant role in decreasing the force exerted on the head. In contrast to the results of this study, findings from previous studies by Donald (2001) and Delaney and Frankovich (2004) suggest that heading techniques are among the most important factors contributing to an increase in force exerted on the head and brain, and thus an increased risk of head and neck injury. This inconsistency could possibly be accounted for by the lack of skill in the participants of this study; based on our evaluation of heading technique, players investigated here scored below average. Therefore, the absence of a relationship between these two variables, i.e., angles of joints and the force exerted on the head, is explicable. Further analyses indicated that there was also no significant correlation between the strength of neck flexor muscles and force when heading *(P* = 0.51); indeed, we observed a negative correlation between these variables. This finding is inconsistent with the results of Delaney and Frankovich (2004) and Mehnert *et al.* (2005). These authors concluded that greater strength of muscles in the neck lessened the force on the head, and consequently limited the seriousness of associated injuries. Regarding our findings, it is possible that the participants in our study had lower than average strength in the neck muscles. For example, the 15 participants used in Tierney *et al.* (2008) had an average muscle strength of 15.88 ± 3.05 kg, while the average muscle strength for players in our study was 12.41 ± 2.95 kg. In order to account for this possibility, we conducted a case study of one of the participants who had a muscle strength of 17 kg, and found that he could bear a force of less than 600 N when heading on all the kicks. Therefore, further studies are needed to assess the relationship between muscle strength and the force exerted on the head. Our results also showed a strong negative correlation between head perimeter and the force exerted on the head when heading free kicks (*P* = 0.00). However, to the best of our knowledge, no other study has been conducted with the same focus with which we can compare our results. Nevertheless, we can account for this negative correlation using several mathematical formulas. According to the pressure formula (P = F/A), the larger the perimeter of the head (A), the less is the pressure (P) exerted on the head, and therefore, the wider the forehead, the less is the pressure exerted on the head when heading.

Our findings also revealed a negative correlation between the percentage of subcutaneous fat and the force exerted on the head when heading free kicks (*P *= 0.02). Again, we found no study with which we could compare our results. However, considering our findings, we can conclude that subcutaneous fat is not only important for thermal insulation (13), it also acts as a shield against impacts to the head. Therefore, soccer players with more subcutaneous fat are less likely to be influenced by impacts to the head, and consequently are less likely to sustain head and neck injuries. Regarding the relationship between body mass and the force exerted on the head when heading, it was found that there was a high negative correlation between the two (*P* = 0.02), which is consistent with the results of other studies (5, 14, 15). Therefore, it can be concluded that because the mass of the ball is fixed, the greater the body mass of the player, the greater the mass of their head, and therefore the greater the momentum of the head will be during heading, resulting in the head being less affected by the force exerted by the ball when heading. We also observed a high negative correlation between the age of the participants and the force exerted on the head (*P *= 0.01), which is consistent with results reported previously (5, 16). This relationship is best explained by the fact that as one gets older, not only do parts of the head grow and the skull becomes thicker, but the muscles of the neck also become stronger and lead to the dispersion of force exerted on the head. It can also be said that as soccer players age, their experience and skills develop and, as a result, techniques such as heading improve, which could lead to lower levels of force being exerted on the head. According to previous findings, a brain injury can result from a force of 400–1000 N (7, 8). This study shows that heading can produce a force of approximately 515–1100 N. Therefore, it seems that when heading, injuries are very likely to happen. However, there is a need for studies that focus on the different factors that can help reduce the force experienced by the head when heading. Our findings showed that anthropometric variables played a more important role in reducing the force exerted on the head. Although body mass and the level of subcutaneous fat were shown to be involved, age and head perimeter seemed to play more important roles in reducing the force.
